# Comparative Analysis of AWJM Performance in FFF-Printed PLA and PLA–CF: Influence of Process Parameters and Cutting Regions

**DOI:** 10.3390/polym18101210

**Published:** 2026-05-15

**Authors:** Pedro F. Mayuet Ares, Lucía Rodríguez-Parada, Sergio de la Rosa, Moises Batista

**Affiliations:** Mechanical Engineering and Industrial Design Department, School of Engineering, University of Cadiz, Avda. de la Universidad de Cádiz, 10, E11519 Puerto Real, Spain; sergio.delarosa@uca.es (S.d.l.R.); moises.batista@uca.es (M.B.)

**Keywords:** abrasive water jet machining, fused filament fabrication, polylactic acid, surface integrity, process parameters

## Abstract

Additive manufacturing by Fused Filament Fabrication (FFF) enables the fabrication of complex polymer components, although limitations in surface quality and dimensional accuracy often require post-processing. Abrasive water jet machining (AWJM) is a non-thermal technique suitable for improving surface integrity in polymers and composites without inducing thermal damage. This study investigates the AWJM performance on FFF-printed polylactic acid (PLA) and carbon-fiber-reinforced PLA (PLA–CF), focusing on the influence of water pressure (WP), traverse feed rate (TFR), and abrasive mass flow rate (AMFR). A full factorial design was implemented, and surface integrity was evaluated through surface roughness (Ra) and kerf taper (T), considering their variation across characteristic cutting regions: initial damage region (IDR), smooth cutting region (SCR), and rough cutting region (RCR). Results show that WP and TFR are the dominant parameters, while AMFR has a limited effect within the studied range. The SCR exhibits the lowest roughness, whereas the RCR shows significant degradation due to energy loss. Both materials present similar behavior, with only minor improvements in PLA–CF. ANOVA confirms that process parameters have a stronger influence than material type, providing useful criteria for AWJM optimization in FFF polymers.

## 1. Introduction

Additive manufacturing (AM) has experienced significant growth in recent decades, establishing itself as a key technology in the development of new design and production paradigms. Unlike conventional manufacturing processes based on material removal, AM enables the generation of parts through the controlled addition of material layer by layer, allowing the production of complex geometries, optimized structures, and high material efficiency [1,2]. This capability has driven its adoption across multiple industrial sectors, facilitating the development of design solutions that were previously unfeasible or economically prohibitive, particularly in fields such as aerospace, automotive, and biomedical engineering [3]. Furthermore, its impact on design optimization and the reduction in costs associated with tooling and traditional processes has supported its integration into various technological domains [1,2].

Among AM technologies, Fused Filament Fabrication (FFF) has become one of the most widely adopted due to its simplicity, accessibility, and versatility. This process is based on the extrusion of molten thermoplastic material, deposited layer by layer following instructions derived from a previously designed CAD model [4]. Its low cost, ease of use, and capability to produce complex geometries have promoted its adoption in both industrial and academic environments [2,5]. In addition, control over parameters such as build orientation, layer thickness, and infill pattern enables the adjustment of the final properties of the part according to application requirements [6,7].

In polymer-based AM, thermoplastic materials represent the most commonly used option due to their ease of processing and stability under repeated thermal cycles [8]. Among them, polylactic acid (PLA) stands out for its good stiffness, ease of printing, and ability to produce parts with acceptable surface quality, as well as its renewable origin. These characteristics make it a suitable material for both rapid prototyping and low- to medium-demand functional applications. Numerous studies have demonstrated its adequate mechanical performance, dimensional stability, and processability, contributing to its widespread use in AM [2,9].

To improve the mechanical performance of these materials, the use of polymer matrix composites has become increasingly common, particularly those reinforced with carbon fiber, which exhibit significant improvements in strength-to-weight ratio [8]. In this context, carbon fiber-reinforced PLA (PLA-CF) combines the processability of thermoplastics with enhanced stiffness and strength, extending its applicability to more demanding industrial environments.

However, parts manufactured by FFF present inherent limitations associated with the process. The successive deposition of layers results in an anisotropic structure, where interlayer bonding constitutes a weak point under mechanical loading [9]. This characteristic, together with the non-instantaneous solidification of the extruded material and its partial fusion with adjacent layers, generates structural discontinuities that affect the mechanical integrity of the material [10]. In addition, surface finish is often poor due to the stair-stepping effect and the influence of manufacturing parameters, which may compromise both dimensional accuracy and in-service performance [6].

These limitations necessitate the use of post-processing operations to improve the final quality of the parts [11]. However, the application of conventional machining methods to polymeric and composite materials presents significant challenges, arising from their heterogeneous nature, thermal sensitivity, and the presence of reinforcements, which may induce defects such as delamination, fiber pull-out, or surface damage [8,12,13]. Furthermore, contact-based processes may generate local deformations, while those involving heat or chemical agents can cause matrix degradation or heat-affected zones (HAZ), compromising material integrity [14,15].

These issues are exacerbated in large-scale additive manufacturing processes, such as Big Area Additive Manufacturing (BAAM), where high deposition rates and large layer thicknesses increase surface roughness, interlayer defects, and geometric deviations [16,17]. Consequently, efficient post-processing techniques are required to meet dimensional and surface quality requirements within competitive time and cost constraints.

In this context, non-conventional machining processes emerge as a promising alternative, as they minimize tool–material contact and associated thermal effects. Among them, abrasive water jet machining (AWJM) stands out for its versatility and its ability to machine a wide range of materials through the erosive action of a high-pressure water jet combined with abrasive particles [18,19]. This process enables progressive material removal without direct contact or the generation of heat-affected zones, which is particularly advantageous for heat-sensitive materials such as polymers and polymer matrix composites [14,20].

AWJM also involves low cutting forces and a high adaptability to different geometries and materials, which has promoted its implementation in industrial sectors such as aerospace and automotive [18,21]. In the case of AM-produced materials, it allows cutting and finishing operations without introducing significant mechanical stresses or altering the material microstructure.

The behavior of the AWJM process is governed by operational parameters such as water pressure (WP), traverse feed rate (TFR), and abrasive mass flow rate (AMFR), which directly influence surface quality, cut geometry, and defect formation [19,22]. In general, water pressure and traverse speed are the dominant factors affecting surface roughness and cut geometry, while abrasive flow rate exhibits a secondary or condition-dependent influence [22,23].

As a result of the interaction between these parameters, the process generates distinct characteristic regions along the material thickness: an initial region (Initial Damage Region, IDR), associated with higher surface disturbance; an intermediate region (Smooth Cutting Region, SCR), where cutting is more stable and surface quality improves; and a final region (Rough Cutting Region, RCR), characterized by increased roughness due to the loss of jet energy [24,25]. The characterization of these regions is essential for understanding and optimizing the process.

Despite its advantages, the application of AWJM to materials manufactured by FFF, particularly composites such as PLA-CF, remains limited. Most existing studies focus on metallic, ceramic, or conventionally manufactured composite materials, with a clear lack of research addressing additively manufactured materials, considering their anisotropic nature and microstructural particularities [14,23].

In polymeric materials, although differences in erosion mechanisms have been identified due to their viscoelastic behavior and lower hardness, available studies have primarily focused on materials produced by conventional processes [18,23]. Similarly, in carbon fiber-reinforced composites, the literature has mainly addressed materials fabricated using traditional techniques, without sufficiently considering those obtained via FFF, where the interaction between structural anisotropy and machining parameters is particularly complex [3,14].

On the other hand, several studies have analyzed the influence of manufacturing parameters on the mechanical and surface properties of PLA and other thermoplastic parts, highlighting the role of variables such as build orientation, layer thickness, and infill pattern in strength, anisotropy, and surface quality [2,26,27]. However, the study of these materials during post-processing operations, and particularly under AWJM, remains limited.

Therefore, a lack of studies is identified that address additive manufacturing and abrasive water jet machining in an integrated manner, particularly for reinforced polymeric materials. While cutting regions have been previously analyzed in conventional materials, their behavior in layered and anisotropic FFF structures remains insufficiently explored. This gap limits the establishment of robust criteria for parameter selection and process optimization, especially when considering both surface and geometric quality variables. Moreover, it restricts a comprehensive understanding of variability across different cutting regions (IDR, SCR, and RCR), including aspects such as the progressive degradation of jet energy in anisotropic materials, the regional sensitivity to process parameters, and the relationship between FFF-induced structure and AWJM response.

In this context, the objective of the present work is to comparatively analyze the behavior of PLA and carbon fiber-reinforced PLA (PLA-CF) specimens manufactured by FFF when subjected to abrasive water jet machining. To this end, the influence of the main process parameters on surface quality—evaluated through average roughness (Ra)—and on cut geometry—characterized by taper angle (T)—is investigated. In addition, the evolution of these parameters across the different cutting regions (IDR, SCR, and RCR) is analyzed in order to identify differences in behavior between both materials.

The ultimate goal is to contribute to a better understanding of the AWJM process applied to polymeric materials manufactured by AM, providing criteria for machining parameter optimization and evaluating the influence of carbon fiber reinforcement on the final cut quality. This knowledge is particularly relevant for industrial applications requiring improved surface finish and dimensional accuracy in parts produced by processes with inherently low initial surface quality, such as BAAM.

## 2. Materials and Methods

### 2.1. Materials

The specimens used in this study were fabricated by FFF using two thermoplastic materials: PLA and carbon fiber-reinforced PLA (PLA-CF). PLA is a widely used polymer in additive manufacturing due to its ease of processing, good dimensional stability, and adequate mechanical properties, making it one of the most commonly employed materials in FFF technologies [2,9].

PLA-CF incorporates approximately 16% short chopped carbon fibers as reinforcement, with the aim of improving stiffness, specific strength, and structural stability, while also modifying its mechanical behavior and its response to machining processes [23].

In both cases, commercial filaments from the brand i3D Tested were used, as the objective of this study is not the development of a new material but the evaluation of the influence of carbon fiber reinforcement on behavior during AWJM post-processing.

Both materials were supplied as filaments with a diameter of 1.75 mm. The mechanical properties of PLA—such as elastic modulus, tensile strength, and elongation at break—are widely reported in the literature. However, these values may vary significantly depending on printing conditions, including build orientation, layer thickness, infill density, and material manufacturer [9,26]. In this study, such variations are considered negligible, assuming representative material behavior under controlled manufacturing conditions.

Furthermore, it should be noted that the internal orientation of the filaments was set to a ±45° weave configuration. This choice was made to reduce the influence of the highly directional anisotropy typically observed in 0/90° or unidirectional layers. Thus, the ±45° arrangement promotes a more balanced mechanical response and prevents preferential crack propagation paths during machining. From a machining perspective, this configuration improves the interaction between the abrasive jet and the material, as the jet continuously intersects filaments with varying orientations, resulting in more uniform erosion behaviour throughout the thickness.

According to manufacturer data, PLA-CF exhibits more than 87% higher stiffness than standard PLA, along with improved impact resistance (Table 1).

### 2.2. Specimen Fabrication

The specimens were manufactured using an FFF system, model Creality CR-10S (Creality, Shenzhen, China), following the standard additive manufacturing process based on layer-by-layer deposition of molten material [4]. The specimen geometry was designed using CAD tools and subsequently processed using slicing CURA software to generate the printer control code (version 5.11).

The manufacturing parameters used during the process are listed in Table 2. It is well established that parameters such as layer thickness, extrusion temperature, deposition speed, and infill pattern directly influence the final quality and mechanical properties of printed parts [6,7]. Therefore, in this study, manufacturer-recommended parameters were used to ensure that specimens were as consistent as possible, so that the variable under investigation was the material rather than the manufacturing conditions.

The resulting specimens exhibited a prismatic geometry with approximate dimensions of 60 × 210 × 6 mm, suitable for subsequent AWJM processing.

### 2.3. AWJM Process

Machining of the specimens was performed using an abrasive water jet machining (AWJM) system, which enables material removal through the action of a high-pressure water jet combined with abrasive particles. In this case, a TCI Cutting BP-C 3020 (TCI cutting, Spain) system was used, capable of operating at ultra-high pressure.

The experiments were conducted using a full factorial design (3^3^), varying the three main process parameters: water pressure (WP), traverse feed rate (TFR), and abrasive mass flow rate (AMFR). These parameters are widely recognized as the most influential factors affecting cutting quality in AWJM processes [19].

The same operating parameters, configurations, and fixturing systems were used for both materials. The parameters employed are listed in Table 3. The standoff distance (SOD) was kept constant at 2.5 mm in all tests, and the abrasive material used was 80 mesh Indian garnet. The final experimental configuration is summarized in Table 4.

**Figure 1:**
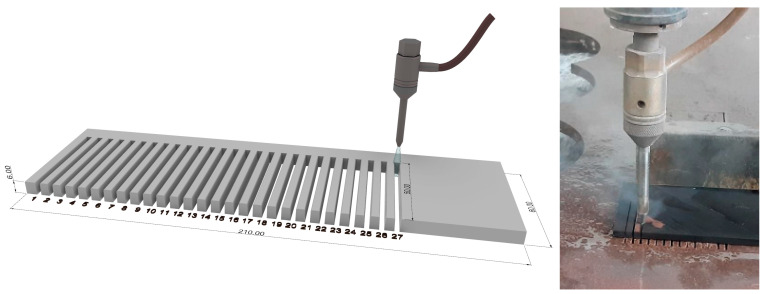
Schematic representation of the slot-cutting strategy.

The tests consisted of cutting 27 slots, resulting from the combination of the selected parameters, each with a length of 50 mm and spaced 6 mm apart (Figure 1). This slot-cutting methodology has been demonstrated to be suitable for characterizing process-related defects and evaluating cutting quality [28]. Moreover, a single slot of this length provides sufficient data for the analysis.

### 2.4. Evaluation of Machining Quality

The evaluation of cutting quality was carried out in two stages.

In the first stage, defect characterization and cut geometry were analyzed using stereoscopic optical microscopy (SOM), specifically with a Nikon SMZ800 (Nikon, Tokyo, Japan) microscope. This allowed for the acquisition of images of the machined surfaces and the identification of defects across the different regions of the specimens. The geometric quality of the cut was evaluated in terms of the taper angle (T). Images were acquired at the jet entry and exit, as well as along a cross-sectional plane perpendicular to the cut, in order to analyze taper and overall cut quality. It should be noted that the microscope was calibrated beforehand using a pixel-micrometre standard at each desired magnification.

The taper angle was calculated using the ImageJ image processing software (version 1.54s). The acquired images were first calibrated, and measurements were subsequently performed. Although different methods exist for calculating taper angle, in this study it was determined based on the difference between the top and bottom kerf widths, using Equation (1):

(1) $$\theta =\tan^{-1}\left(\frac{{W_{T}-W}_{E}}{2\cdot e}\right)$$

where

- θ:Taperangle;
- $W_{T}:Topkerfwidth$;
- $W_{E}:Bottomkerfwidth$;
- e:Specimenthickness.

In the second stage, surface quality was analyzed in terms of arithmetic average roughness (Ra), according to the UNE-EN ISO 4288 standard [29], using a calibrated Mahr Perthometer PGK 120 (Mahr, Germany) contact profilometer. Access to the interior of the slots required a longitudinal cut to be performed.

**Figure 2:**
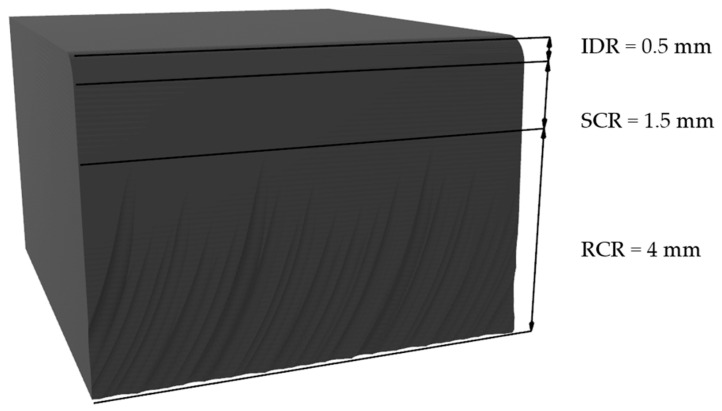
Schematic representation of the cutting regions used for roughness analysis.

Ra is a commonly used parameter for characterizing surface quality in AWJM processes [28]. The analysis was conducted considering the different characteristic cutting regions: the Initial Damage Region (IDR), the Smooth Cutting Region (SCR), and the Rough Cutting Region (RCR), which exhibit distinct behaviors depending on the jet–material interaction. To ensure consistency in region identification, ranges were defined based on previous studies [25,30], as illustrated in Figure 2.

### 2.5. Statistical Analysis

To evaluate the influence of process parameters on the response variables, a statistical analysis based on analysis of variance (ANOVA) was performed. This method allows for the identification of the significance of each factor and their interactions with respect to the studied variables, facilitating the interpretation of the obtained results.

The statistical analysis was applied independently to each material and to each cutting region, enabling comparison of the behavior of PLA and PLA-CF under different machining conditions.

Generative artificial intelligence (GenAI) tools were used for text analysis, discussion enhancement, and figure generation.

## 3. Results and Discussion

The analysis of the results was conducted by combining a qualitative evaluation of the machined surfaces with a quantitative study of the cutting quality parameters. First, a visual analysis was performed using stereoscopic microscopy in order to identify the main defect formation mechanisms and the evolution of the cutting process throughout the material thickness. Subsequently, the influence of the process parameters—water pressure, traverse feed rate, and abrasive mass flow rate—on surface roughness (Ra) and taper angle (T) was analyzed, considering both their individual behavior and their overall effect through statistical analysis. This approach makes it possible to establish a direct relationship between the observed morphology and the quantitative results, thus facilitating an integrated interpretation of the abrasive water jet machining process in FFF-manufactured materials.

### 3.1. Qualitative Analysis of Defects by Stereoscopic Microscopy

The qualitative analysis of the machined surfaces was carried out by stereoscopic optical microscopy, which made it possible to identify the main morphological features generated during the abrasive water jet machining (AWJM) process. This type of analysis is particularly useful for understanding the evolution of the process along the material thickness and for establishing a direct relationship between the observed morphology and the applied machining conditions.

**Figure 3:**
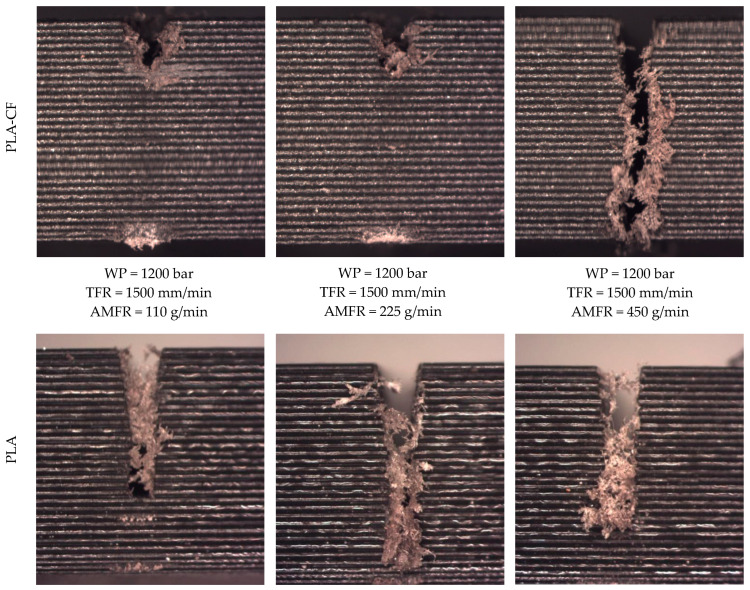
Results of PLA and PLA-CF tests in which the cut was not completed.

**Figure 4:**
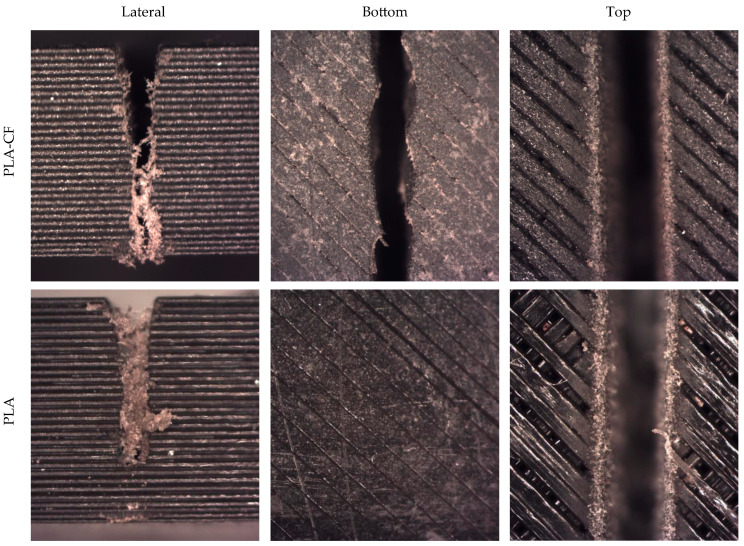
Results of the test performed at WP = 1200 bar, TFR = 1000 mm/min, and AMFR = 110 g/min on PLA and PLA-CF, in which the cut was not completed.

The first aspect to highlight is that the process parameters exert a decisive influence on the cutting process. In fact, in tests 25, 26, and 27, the cut was not completed, as can be seen in Figure 3. These tests correspond to the configuration with the lowest working pressure and the highest traverse speed. A similar behavior was observed in test 22, which was only partially completed, with only some regions being cut; in this case, the traverse speed was slightly lower, but the reduction in abrasive flow rate resulted in incomplete penetration (Figure 4). This occurred in both studied materials.

**Figure 5:**
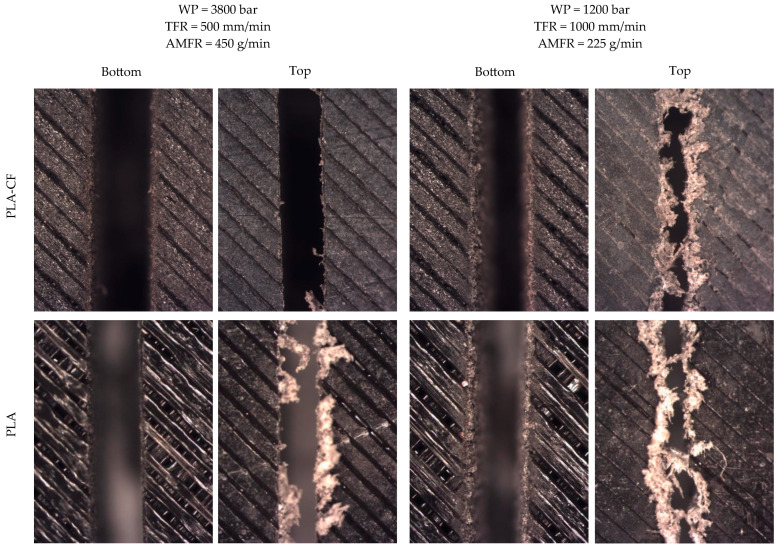
Results of PLA and PLA-CF tests at the jet entry and exit regions.

Another significant finding is that, although it could initially be assumed that the reinforced material, due to its improved mechanical properties, would be more difficult to cut, the results show similar or even less favorable behavior in the reinforced material. Therefore, the reinforcement does not appear to act as a controlling factor in the process. On the other hand, the entry regions are similar regardless of the cutting parameters, whereas the exit regions clearly exhibit different behaviors depending on the aggressiveness of the process conditions, understood here as those involving higher pressure, lower traverse speed, and higher abrasive flow rate (Figure 5). In the entry region, only slight abrasion is observed, which is more evident in tests performed under less aggressive conditions. However, in the exit region, a pronounced striation or zigzag pattern can be observed, which is characteristic of a loss of cutting capability of the jet due to the decrease in its energy throughout the material thickness. This phenomenon has been widely documented in AWJM and is associated with the progressive reduction in particle velocity and process instability in the final cutting region [22].

It is also noteworthy that the burrs observed are smaller in PLA-CF than in PLA. This behavior could be related to the presence of fibrous reinforcement, which promotes more brittle fracture mechanisms and greater material fragmentation during the erosion process, thereby reducing plastic deformation at the cut edges. This type of response has been described in reinforced composite materials, where the matrix–reinforcement interaction modifies material removal mechanisms and limits burr formation compared to unreinforced polymeric materials [14,23]. A similar behavior has also been reported in other composite materials, such as metal matrix composites [31].

**Figure 6:**
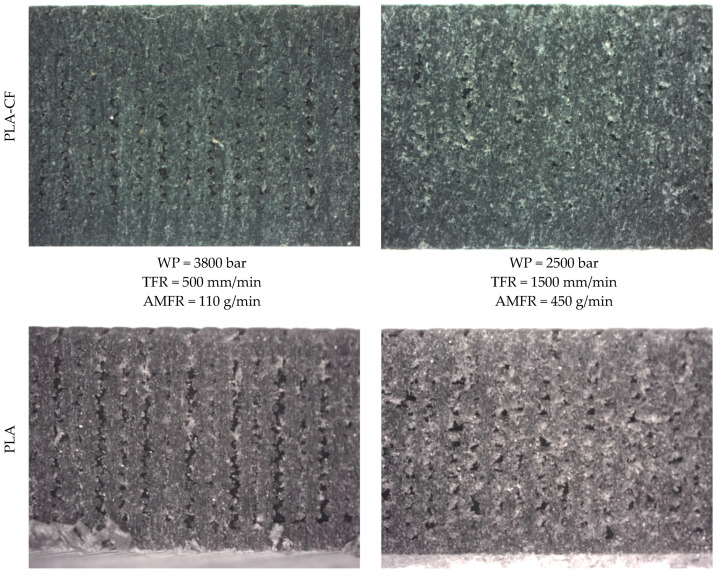
Results of PLA and PLA-CF tests in the cutting region.

As previously mentioned, the formation of an Initial Damage Region (IDR), a Smooth Cutting Region (SCR), and a Rough Cutting Region (RCR) is characteristic of the AWJM process, as described in the literature [30]. These regions exhibit clearly differentiated morphologies, reflecting the different interaction regimes between the jet and the material. However, in these materials, it is difficult to determine precisely where each region appears (Figure 6). A first more eroded zone producing rounded features is clearly observed, together with a final zone where quality deteriorates, as evidenced by cut taper, but these regions are not sharply defined. In this sense, the dominant mechanism of material removal is surface plastic deformation and micro-ploughing, due to its viscoelastic behaviour. The impact of the abrasive particles initially causes material displacement and grooving, whilst fracture and micro-cutting only occur secondarily due to damage accumulation and the propagation of microcracks. Consequently, the erosion of PLA is governed primarily by deformation processes, with a minor contribution from brittle cutting mechanisms.

Figure 6 shows representative examples of the cutting regions for both materials. The comparison between PLA and PLA-CF reveals that, from a qualitative point of view, both materials exhibit similar behavior in terms of the formation of the different cutting regions. Nevertheless, PLA-CF shows a slight tendency toward more homogeneous surfaces, which could be related to the presence of reinforcement and its influence on the material erosion mechanism. In addition, in both cases, particles with different coloration can be observed, which could be associated with embedded abrasive grains that may become lodged in the material interface due to a ploughing effect.

The visual analysis confirms that the AWJM process generates a stratified cutting structure in both materials, promoted by the deposited material layers, and that the differences between PLA and PLA-CF are subtle from a morphological standpoint. These qualitative results provide the basis for the subsequent quantitative analysis of surface roughness and taper angle, allowing the experimental results to be interpreted as a function of process evolution throughout the material thickness.

### 3.2. Influence of Process Parameters on Surface Roughness (Ra)

The marginal analysis of the study variables is first presented, followed by the overall analysis of the three process parameters.

#### 3.2.1. Influence of Water Pressure (WP)

**Figure 7:**
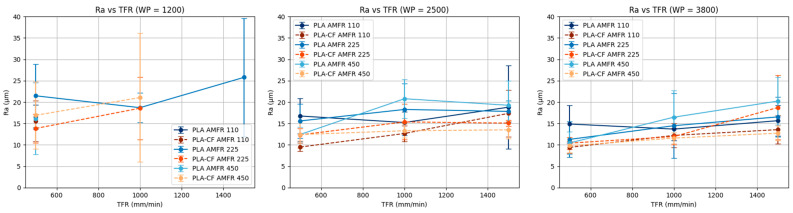
Marginal analysis of Ra at constant water pressure.

When water pressure (WP) is kept constant, surface roughness (Ra) tends to increase with increasing traverse feed rate (TFR) in most parameter combinations, especially for PLA (Figure 7). This behavior is consistent with the physics of the AWJM process, since increasing traverse speed reduces the interaction time between the jet and the material, decreasing energy transfer and consequently deteriorating cut quality. This effect has been widely documented, showing an increase in surface roughness and cutting irregularities as traverse speed increases due to the reduced ability of the jet to maintain a stable erosion regime [18,22].

Moreover, PLA exhibits greater sensitivity to TFR, as evidenced by steeper slopes compared with PLA-CF. In contrast, PLA-CF shows a more stable response, with smoother variations in Ra as TFR changes, suggesting better resistance to surface deterioration under more demanding cutting conditions.

Finally, the lack of parallelism among the curves corresponding to different AMFR levels indicates the presence of significant interactions between TFR and AMFR. These interactions are particularly noticeable at low WP levels, where the process appears less stable and more dependent on parameter combinations.

#### 3.2.2. Influence of Traverse Feed Rate (TFR)

**Figure 8:**
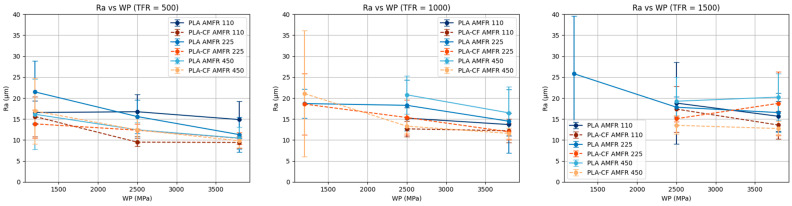
Marginal analysis of Ra at constant traverse feed rate.

When traverse feed rate is kept constant, surface roughness (Ra) generally decreases as water pressure increases (Figure 8). This effect is more evident in PLA, where increasing pressure clearly improves surface quality, probably due to the greater erosive capability of the jet.

In the case of PLA-CF, although the same decreasing trend is observed, the behavior is more uniform and less sensitive to changes in WP, reinforcing the idea that this material exhibits greater stability against process variations. Differences among AMFR levels are also less pronounced than in PLA.

Likewise, interactions between water pressure (WP) and abrasive mass flow rate (AMFR) can be identified, especially at high traverse feed rates, where some curves show changes in trend or convergence. This behavior indicates that the beneficial effect of increasing pressure may depend on the amount of abrasive available, demonstrating that the process parameters do not act independently. Various studies have highlighted the existence of significant interactions between AWJM parameters, where specific combinations of pressure, abrasive flow rate, and traverse speed determine process efficiency and cutting quality [22,32].

#### 3.2.3. Influence of Abrasive Mass Flow Rate (AMFR)

**Figure 9:**
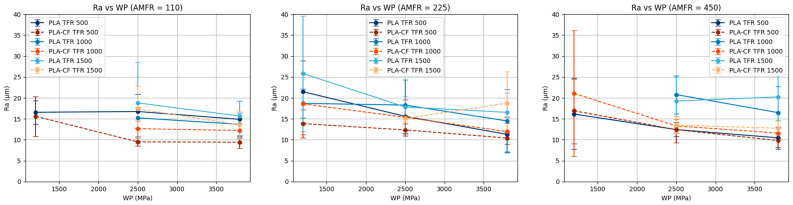
Marginal analysis of Ra at constant abrasive mass flow rate.

When abrasive mass flow rate is kept constant (Figure 9), the analysis shows that water pressure remains a key factor, with a general trend toward lower Ra values as WP increases, especially in PLA-CF. This behavior is consistent with improved cutting efficiency as jet energy increases.

On the other hand, PLA shows greater result dispersion, with behavior that is not always monotonic, particularly at high AMFR values. In these cases, the effect of WP is less clear, indicating greater complexity in the interaction between process parameters.

Finally, this analysis reveals a strong interaction between water pressure (WP) and traverse feed rate (TFR), since the curves corresponding to different TFR levels are not parallel and even intersect within certain operating ranges. This behavior confirms that the effect of pressure strongly depends on traverse speed, evidencing a significant interaction between both parameters. In this regard, several studies have shown that cutting quality in AWJM is governed by the combined action of process parameters, requiring joint optimization in order to achieve efficient and stable machining conditions [22,32].

#### 3.2.4. Overall Analysis

**Figure 10:**
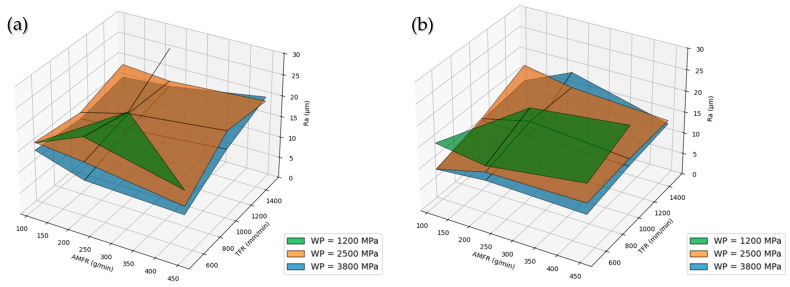
Three-dimensional representation of Ra as a function of water pressure for each material: (**a**) PLA; (**b**) PLA-CF.

When the data are analyzed as a whole, water pressure (WP) emerges as the parameter with the greatest influence on surface roughness in the AWJM process. The obtained results show a clear decrease in Ra values as pressure increases, for both PLA and PLA-CF (Figure 10).

The surface roughness obtained in the AWJM process shows a clear dependence on the process parameters, with water pressure and traverse feed rate being the most influential factors, whereas abrasive mass flow rate has a secondary effect. In particular, it has been shown that water pressure controls the energy available for erosion and the cutting capability, while traverse feed rate governs the interaction time between the jet and the material, thereby determining the resulting surface quality. In contrast, once an adequate threshold has been reached, abrasive flow rate has a less significant influence compared with these main parameters [18,22,30].

Increasing water pressure leads to a reduction in surface roughness, which is associated with the increase in jet kinetic energy and a more effective interaction between abrasive particles and the material. This increase in energy promotes a more stable and uniform erosion process, improving cutting capability and reducing irregularities generated during machining. This behavior has been widely documented, showing that higher pressures increase process efficiency and surface quality due to the higher velocity of abrasive particles and their greater penetration capability into the material [22,32].

In contrast, increasing traverse feed rate produces an increase in surface roughness values in both materials due to the reduction in interaction time between the jet and the surface. At higher traverse speeds, the erosion process becomes less effective because the energy transfer from the abrasive particles to the material decreases, resulting in greater surface irregularities. This behavior has been widely observed in AWJM, where traverse feed rate is identified as a critical parameter that negatively affects surface quality when increased beyond certain values [18,22,30].

Regarding abrasive mass flow rate, its influence is limited within the studied range. Once a sufficient number of particles is present in the jet, the process no longer depends on abrasive availability and becomes mainly controlled by jet energy and interaction time, so additional increases do not translate into significant improvements in surface quality [22,32]. In addition, excessive abrasive flow may reduce process efficiency by lowering the individual impact energy of the particles.

The comparison between materials indicates that both PLA and PLA-CF show similar trends with respect to variations in the process parameters. However, PLA-CF exhibits slightly lower surface roughness values in some cases, which may be related to the presence of fibrous reinforcement and the higher stiffness of the material, which increase resistance to material removal during the erosion process. In fiber-reinforced composites, the interaction between matrix and reinforcement modifies material removal mechanisms, in some cases promoting a more stable response to abrasive particle impact and improved surface quality [14,23].

Water pressure is identified as the determining process parameter, as it controls the energy available for erosion and acts as the main factor in reducing surface roughness. Increasing pressure implies an increase in abrasive particle velocity and, therefore, in their penetration and material removal capability, promoting a more efficient and uniform cut [22]. Traverse feed rate, in turn, governs the interaction time between the jet and the material, so high TFR values reduce effective energy transfer and compromise process stability, leading to increased surface roughness, especially under critical machining conditions [32]. In contrast, abrasive mass flow rate has a limited influence on roughness within certain operating ranges, being outweighed by the combined effect of jet energy and interaction time, which indicates that its contribution to process optimization is secondary compared with other key parameters [22].

#### 3.2.5. Analysis by Cutting Regions

**Figure 11:**
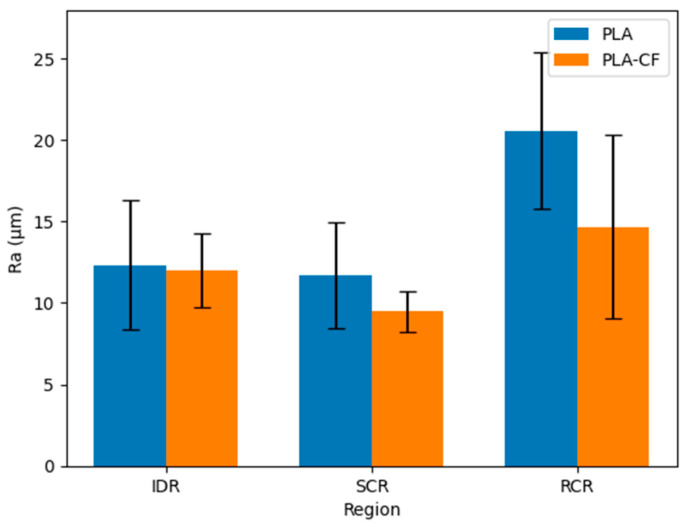
Average surface roughness (Ra) for PLA and PLA-CF across the different AWJM cutting regions (IDR, SCR and RCR).

The analysis of surface roughness by cutting region makes it possible to understand the evolution of the AWJM process throughout the material thickness. As observed, the cut exhibits three differentiated zones—the Initial Damage Region (IDR), Smooth Cutting Region (SCR), and Rough Cutting Region (RCR)—associated with different jet–material interaction regimes, as widely described in the literature [18,30]. Figure 11 shows the average surface roughness (Ra) together with the standard deviation for each cutting region. The SCR exhibits both the lowest roughness values and the lowest variability, indicating a more stable erosion regime, whereas greater dispersion is observed in the IDR and RCR.

**Figure 12:**
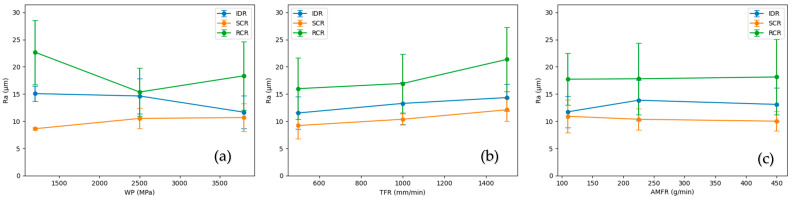
Influence of process parameters on surface roughness (Ra) for different cutting regions: (**a**) water pressure (WP), (**b**) traverse feed rate (TFR), and (**c**) abrasive mass flow rate (AMFR).

As shown in Figure 12, the influence of process parameters on surface roughness varies depending on the cutting region, with traverse feed rate (TFR) having the greatest effect, especially in the RCR.

The Smooth Cutting Region (SCR) systematically exhibits the lowest roughness values, constituting the highest-quality cutting zone. In this region, a stable erosion regime predominates, in which the combination of jet energy and interaction time allows for more uniform material removal. For this reason, the influence of process parameters—especially water pressure (WP) and traverse feed rate (TFR)—is most clearly observed here.

In contrast, the Rough Cutting Region (RCR) shows a significant increase in roughness, associated with the progressive loss of jet energy as it passes through the material, which promotes the appearance of striations and discontinuities. In the Initial Damage Region (IDR), roughness exhibits greater dispersion due to the chaotic nature of the initial jet impact, where the high available energy generates a less controlled erosion process [22,30].

The influence of process parameters varies according to the region. Increasing water pressure reduces roughness throughout the thickness, with a more pronounced effect in the SCR. Traverse feed rate, in turn, has a particularly relevant influence in this region, where the process is more sensitive to interaction time: at low speeds, more homogeneous surfaces are obtained, whereas increasing TFR leads to greater irregularity. In the RCR, this effect intensifies due to the combination of lower residual energy and shorter exposure time [18,22,30].

In contrast, abrasive mass flow rate (AMFR) shows a limited influence in all regions. In the SCR, hardly any variations are observed, whereas in the RCR some dispersion is present without a defined trend, and in the IDR its effect is masked by the initial jet impact [22].

The comparison between materials indicates that both PLA and PLA-CF present a similar roughness distribution in response to process parameter variation, suggesting that the process is governed more by jet conditions than by microstructural differences. However, PLA-CF shows slightly lower roughness values in some cases, especially in the SCR, which may be attributed to the fibrous reinforcement and the increased stiffness, favoring greater resistance to localized erosion [23]. Nevertheless, these differences are not sufficiently significant to establish a clearly differentiated behavior, in agreement with previous studies in which AWJM parameters prevail over material properties within certain ranges [22].

Overall, the results confirm that cut surface quality is strongly conditioned by the evolution of jet energy throughout the thickness, with the SCR being the most representative region of the stable cutting regime.

### 3.3. Influence of Process Parameters on the Taper Angle (T)

The taper angle (T) was used as a parameter to evaluate the geometric accuracy of the cut, being directly related to the variation in slot width between the jet entry and exit. The obtained results show that taper behavior follows trends similar to those observed for surface roughness, although with lower overall sensitivity to process parameters.

In general, both PLA and PLA-CF exhibit similar taper values, with only slight differences that are not practically decisive. The mean values obtained are within the range previously reported in the literature, with a slight reduction in the case of PLA-CF.

#### 3.3.1. Influence of Water Pressure (WP)

**Figure 13:**
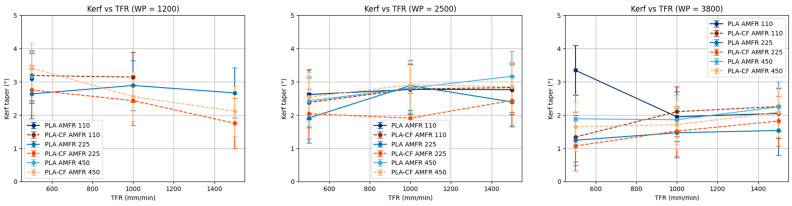
Marginal analysis of kerf taper at constant water pressure.

When water pressure (WP) is kept constant, kerf taper generally shows an increasing trend with increasing traverse feed rate (TFR), especially in PLA-CF (Figure 13). This behavior may be attributed to the reduction in jet–material interaction time at higher speeds, which decreases the ability to maintain uniform cutting throughout the material thickness and promotes groove taper.

In the case of PLA, although the trend in some cases also points to an increase in kerf taper with TFR, greater dispersion and variability are observed in the curves, with behavior that is not strictly monotonic. This suggests greater sensitivity of the material to cutting conditions, as well as a potentially significant influence of parameter interactions.

Likewise, the lack of parallelism among the curves corresponding to different AMFR levels indicates the presence of relevant interactions between TFR and AMFR. These interactions are particularly evident at intermediate and low WP levels, where small variations in abrasive flow rate noticeably modify the evolution of kerf taper with traverse feed rate.

#### 3.3.2. Influence of Traverse Feed Rate (TFR)

**Figure 14:**
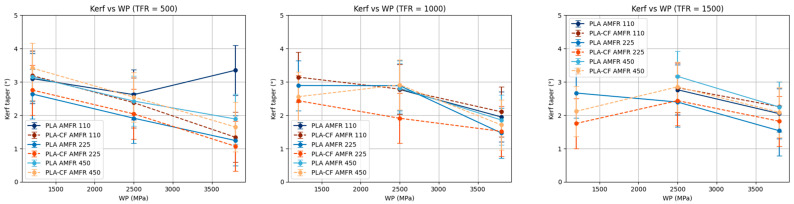
Marginal analysis of kerf taper at constant traverse feed rate.

For constant traverse feed rate (TFR), kerf taper consistently decreases as water pressure (WP) increases in both materials (Figure 14). This result is consistent with the increase in jet kinetic energy at higher pressures, which enables more efficient penetration and a reduction in cut divergence.

PLA-CF shows more uniform and predictable behavior, with a clear decreasing trend in kerf taper as WP increases. In contrast, PLA exhibits slight deviations from this trend in some cases, especially at high AMFR levels, where slope changes or local stabilizations may be observed.

Moreover, the differences among AMFR levels reveal the existence of interactions between WP and AMFR, particularly at intermediate TFR values. In these cases, the beneficial effect of increasing pressure may be modulated by the amount of abrasive, indicating that kerf taper optimization does not depend exclusively on a single parameter, but on the combination of several.

#### 3.3.3. Influence of Abrasive Mass Flow Rate (AMFR)

**Figure 15:**
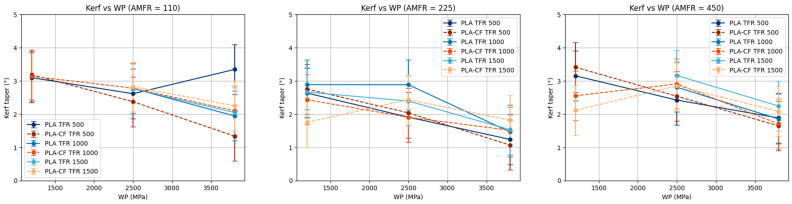
Marginal analysis of kerf taper at constant abrasive mass flow rate.

When abrasive mass flow rate (AMFR) is kept constant, water pressure (WP) is again confirmed as a determining factor, with a general tendency for kerf taper to decrease as WP increases in both materials (Figure 15). This effect is particularly marked in PLA-CF, where the curves exhibit more clearly defined and consistent slopes.

In the case of PLA, although the overall decreasing trend remains, greater result dispersion and some nonlinearity are observed, especially at high TFR values. This suggests that material behavior is influenced in a more complex way by the interaction between process parameters.

Finally, the separation and intersection of the curves associated with different TFR levels provide evidence of a significant interaction between WP and TFR. In particular, the effect of pressure on kerf taper is not uniform for all traverse feed rates, reinforcing the need for a multifactorial approach in AWJM process optimization.

#### 3.3.4. Overall Analysis

Water pressure (WP) is identified as the most influential parameter affecting the kerf taper angle. Increasing WP leads to a reduction in T, which translates into a straighter cut with less divergence between entry and exit. This behavior is associated with the increase in jet energy, which allows greater cutting capability to be maintained throughout the entire material thickness, reducing the loss of efficiency in the lower cutting region.

The influence of pressure is consistently observed in both materials, indicating that taper control is governed mainly by jet energy rather than by the nature of the material.

Traverse feed rate (TFR), in turn, has a less pronounced influence on the cut taper angle. In general, increasing TFR tends to slightly increase taper as a consequence of the reduced interaction time between the jet and the material, which limits the jet’s ability to maintain uniform cutting throughout the thickness. However, since taper is mainly determined by the progressive loss of jet energy as it penetrates the material, its dependence on traverse feed rate is secondary compared with the influence of water pressure [18,22]. In this sense, cut geometry is fundamentally governed by the residual jet energy and its penetration capability, whereas TFR acts as an adjustment parameter that modulates, but does not dominate, process behavior.

As for abrasive mass flow rate (AMFR), its influence on taper angle is limited within the studied range. Once a sufficient concentration of abrasive particles is reached in the jet, the process becomes mainly controlled by jet energy and its capacity to sustain erosion throughout the material thickness. Under these conditions, additional variations in AMFR do not generate significant changes in cut geometry, since process efficiency depends more on particle velocity and jet stability than on the available abrasive quantity [18,22].

The comparison between materials shows that both PLA and PLA-CF exhibit very similar behavior in terms of taper angle, with only minor differences. Although PLA-CF tends to show slightly lower values, these variations do not allow a clear improvement in geometric cutting accuracy to be established, reinforcing the idea that the AWJM process is dominated by operating parameters.

**Figure 16:**
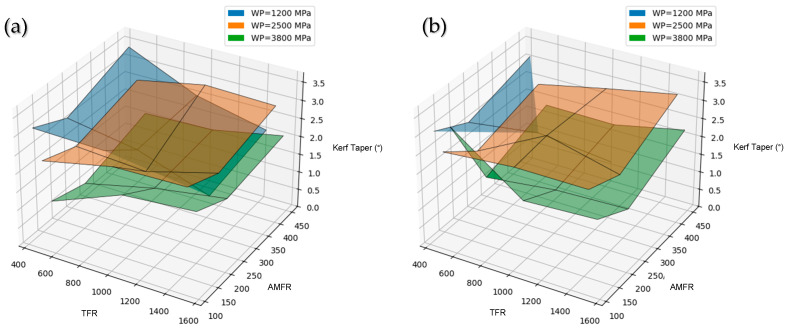
Three-dimensional representation of kerf taper (T) as a function of traverse feed rate (TFR) and abrasive mass flow rate (AMFR) for different water pressure levels (WP) in (**a**) PLA and (**b**) PLA-CF.

This behavior is illustrated in Figure 16, which shows the interaction between process parameters through three-dimensional surfaces, where the combined influence of traverse feed rate and abrasive mass flow rate is observed for different pressure levels.

### 3.4. Global Statistical Analysis (ANOVA)

To quantitatively validate the trends observed in the previous sections, an analysis of variance (ANOVA) was performed on the surface roughness (Ra) and taper angle (T) values, considering water pressure (WP), traverse feed rate (TFR), and abrasive mass flow rate (AMFR) as input factors.

#### 3.4.1. ANOVA for Roughness

For PLA-CF (Table 5), roughness is dominated by traverse feed rate (TFR), which is the only factor showing statistical significance at the 5% level. Water pressure and abrasive mass flow rate do not show a significant main effect within the analyzed experimental domain, and no relevant interactions between factors are detected. In physical terms, this indicates that, for this material, surface quality depends mainly on the effective interaction time between the abrasive jet and the material, while the system responds relatively robustly to simultaneous variations in WP and AMFR.

From a mechanistic point of view, this result suggests that PLA-CF exhibits a more stable cutting response, in which process kinematics control roughness more clearly than jet energy or abrasive load. The absence of significant interactions is consistent with the more regular behavior observed in the marginal plots.

For PLA, both water pressure (WP) and traverse feed rate (TFR) exert a significant influence on surface roughness. AMFR does not appear as a significant main effect, although the TFR × AMFR interaction is very close to the conventional significance threshold, suggesting a combined dependence between traverse speed and abrasive load.

Physically, this behavior indicates that PLA is more sensitive than PLA-CF to both the available jet energy and the interaction time. The near-significance of the TFR × AMFR interaction suggests that abrasive efficiency is not independent of traverse feed rate, but that both factors may act jointly in the generation of striations and in the degradation of surface finish.

The material factor exhibits a highly significant effect on roughness, confirming that PLA and PLA-CF cannot be considered equivalent from the standpoint of surface finish (Table 6). In addition, WP and TFR remain significant in the combined analysis, whereas AMFR does not reach statistical significance.

It is particularly important that the Material × WP, Material × TFR, and Material × AMFR interactions are not significant. This indicates that, although the mean roughness level changes significantly between materials, the overall direction of the parameter effects is comparable in both cases within the considered domain. In other words, the material shifts the response level, but does not radically alter the factorial behavior structure.

Table 7 shows the ANOVA of the influence of process parameters on Ra for each material and cutting region. For PLA-CF, cutting region is clearly the dominant factor, with very high significance. This result confirms that surface roughness is strongly conditioned by the position within the thickness, evidencing the progressive degradation of the cutting process.

Among the process parameters, only traverse feed rate (TFR) shows a significant effect, in agreement with the previous analyses that did not consider cutting region. The Region × TFR interaction is also significant, indicating that the effect of traverse speed is not uniform throughout the thickness, but intensifies particularly in the lower cutting regions.

From a physical standpoint, this suggests that PLA-CF maintains relatively stable behavior in the upper region, whereas in the lower region the loss of jet energy amplifies the effect of traverse feed rate on roughness.

For PLA, cutting region is again the most influential factor, with even higher significance than that observed in PLA-CF, indicating greater sensitivity of the material to process degradation along the thickness.

Unlike PLA-CF, both water pressure (WP) and traverse feed rate (TFR) are significant factors, confirming a more complex behavior that is more dependent on process energy. In addition, the Region × WP and Region × TFR interactions are also significant, showing that the effect of these parameters varies markedly among the different cutting regions.

This result indicates that, in PLA, surface quality depends not only on the position within the thickness, but also on how the process parameters modify jet evolution along that depth. In particular, deterioration in the lower region is more pronounced and more sensitive to changes in pressure and traverse speed.

The combined analysis confirms that both material and region are highly significant factors in surface roughness (Table 8), with region being the factor with the greatest weight in the model. The significance of the Material × Region interaction demonstrates that the evolution of roughness along the thickness depends on the material; that is, PLA and PLA-CF do not exhibit the same degradation pattern.

In addition, traverse feed rate remains significant and the Region × TFR interaction appears again, reinforcing the idea that process kinematics particularly control deterioration at greater depth.

From a physical perspective, these results indicate that, although both materials follow the same general trend, namely worsening with depth, PLA exhibits more pronounced degradation and greater dependence on process parameters. In contrast, PLA-CF shows more stable behavior and lower sensitivity to interactions.

#### 3.4.2. ANOVA for Kerf Taper

Table 9 presents the analysis of taper angle. For PLA-CF, kerf taper is strongly governed by the three main factors, with a clearly dominant role of water pressure, followed by TFR and AMFR. The magnitude of the F-statistic associated with WP shows that jet energy is the most determining mechanism in reducing cut taper.

The absence of significant interactions indicates that, in this material, each parameter acts in an essentially additive manner on cut geometry. From a physical standpoint, this suggests that PLA-CF responds regularly to increases in energy, modifications in traverse feed rate, and abrasive load, without strong couplings between variables appearing within the studied process window.

For PLA, kerf taper depends significantly on WP and AMFR, whereas TFR does not show a significant main effect. This indicates that cut taper is controlled mainly by the combination of jet energy and abrasive availability, rather than by traverse feed rate itself.

Although the interactions do not reach statistical significance at the 5% level, the F-values associated with WP × TFR and TFR × AMFR are appreciable, suggesting a certain degree of complexity in the geometric response of PLA. From a physical point of view, this may be interpreted as greater sensitivity of this material to changes in the erosion regime, even though the main dependence remains associated with pressure and abrasive flow rate.

Unlike roughness, the material factor does not present a significant effect on kerf taper in the combined analysis (Table 10). This indicates that, within the analyzed conditions, cut taper is governed mainly by the process parameters, especially water pressure and, to a lesser extent, abrasive mass flow rate.

The absence of significant interactions between material and parameters confirms that the effect of WP, TFR, and AMFR on kerf taper is essentially comparable in PLA and PLA-CF. Therefore, groove geometry appears to be more closely linked to the general physics of the abrasive jet than to the specific nature of the material, at least within the studied factor range.

Overall, the ANOVA results validate the conclusions obtained from the experimental analysis, confirming that water pressure and traverse feed rate are the key parameters for controlling cut quality, whereas abrasive mass flow rate has a limited effect within the studied range of conditions.

### 3.5. Future Work

Future work should focus on expanding the understanding of abrasive water jet machining (AWJM) applied to additively manufactured polymers and their composites. In particular, the influence of build orientation and raster angle on machinability deserves further investigation, given the inherent anisotropy of fused filament fabrication (FFF) materials. Additionally, a more detailed analysis of erosion mechanisms, including the interaction between abrasive particles and fiber-reinforced structures, would provide deeper insight into material removal processes.

Moreover, future studies could explore a wider range of process parameters and materials, including different polymer matrices and reinforcement types, as well as the optimization of AWJM conditions for improved surface quality and dimensional accuracy. The integration of advanced characterization techniques, such as high-resolution microscopy or in situ monitoring, may also contribute to a better understanding of jet–material interaction. Finally, the development of predictive models linking process parameters, material structure, and surface integrity represents a promising direction for advancing hybrid manufacturing applications.

## 4. Conclusions

This work analyzed the behavior of the abrasive water jet machining (AWJM) process applied to PLA and carbon fiber-reinforced PLA (PLA-CF) specimens manufactured by FFF, evaluating the influence of the main process parameters on surface roughness and cut geometry.

The obtained results allow the following main conclusions to be drawn:

- The AWJM process generates a differentiated cutting structure composed of three regions (IDR, SCR, and RCR), whose morphology and surface quality depend on the evolution of jet energy throughout the material thickness.
- Water pressure (WP) is identified as the most influential parameter, as it controls the energy available for erosion and allows both surface roughness and taper angle to be reduced.
- Traverse feed rate (TFR) has a significant influence, acting as the controlling parameter for the interaction time between the jet and the material. Increasing TFR leads to higher roughness and a slight degradation in cut quality.
- Abrasive mass flow rate (AMFR) has a limited effect within the studied range of conditions, indicating that, once a sufficient particle concentration is reached, its influence on cut quality becomes secondary.
- Both PLA and PLA-CF exhibit similar behavior under the AWJM process, without significant differences in the overall response. Nevertheless, PLA-CF shows a slight improvement in surface quality under certain conditions, especially in the Smooth Cutting Region.
- Cut quality is strongly conditioned by the combination of jet energy and interaction time, which are the key factors for process optimization in FFF-manufactured materials.

Regarding the influence of material, the results indicate that both PLA and PLA-CF exhibit very similar behavior under the AWJM process. This suggests that, under the studied conditions, the erosion mechanism is mainly governed by the interaction between the jet and the polymeric matrix, while the contribution of carbon fiber reinforcement is secondary. However, the slight improvement observed in PLA-CF, particularly in the Smooth Cutting Region, may be related to greater material resistance to localized erosion, which would promote more uniform material removal.

Overall, the obtained results contribute to a better understanding of the AWJM process applied to polymeric and composite materials manufactured by additive manufacturing, providing useful criteria for parameter selection and for improving machining quality.

## Figures and Tables

**Table 1:** Parameters used in specimen fabrication.

Parameters	PLA	PLA-CF
*Modulus of elasticity* (MPa)	3500	6560
*Impact resistance* (kJ/m^2^)	≤5	5.5–6.6

**Table 2:** Parameters used in the manufacture of the test specimens.

Parameters	PLA	PLA-CF
*Extrusion temperature* (°C)	210	210
*Extrusion speed* (mm/s)	50	50
*Layer thickness* (mm)	0.2	0.2
*Wall thickness* (mm)	0.8	0.8
*Fill percentage* (%)	100	100

**Table 3:** Parameters used in the post-processing of the specimens using AWJM.

Parameters	Level 1	Level 2	Level 3
*Water jet pressure* (bar)	1200	2500	3800
*Traverse Feed Rate* (mm/min)	500	1000	1500
*Abrasive mass flow* (g/min)	110	225	450

**Table 4:** Experimental design based on parameter combinations.

Test	WP (bar)	TFR (mm/min)	AMFR (g/min)
1	3800	500	110
2	3800	500	225
3	3800	500	450
4	3800	1000	110
5	3800	1000	225
6	3800	1000	450
7	3800	1500	110
8	3800	1500	225
9	3800	1500	450
10	2500	500	110
11	2500	500	225
12	2500	500	450
13	2500	1000	110
14	2500	1000	225
15	2500	1000	450
16	2500	1500	110
17	2500	1500	225
18	2500	1500	450
19	1200	500	110
20	1200	500	225
21	1200	500	450
22	1200	1000	110
23	1200	1000	225
24	1200	1000	450
25	1200	1500	110
26	1200	1500	225
27	1200	1500	450

**Table 5:** ANOVA of the influence of process parameters on Ra for each material.

	PLA-CF		PLA	
*Effect*	*F*	*p*	*F*	*p*
*WP*	1.621	0.2719	14.530	**0.0189**
*TFR*	7.218	**0.0471**	18.032	**0.0100**
*AMFR*	1.291	0.3694	0.871	0.4853
*WP × TFR*	0.238	0.7983	1.007	0.4423
*WP × AMFR*	0.126	0.8850	0.423	0.6816
*TFR × AMFR*	0.586	0.6912	6.095	0.0540

**Table 6:** ANOVA of the influence of material on Ra.

*Effect*	*F*	*p*
*Material*	21.462	**0.000276**
*WP*	7.006	**0.0176**
*TFR*	14.735	**0.000235**
*AMFR*	0.341	0.7163
*Material × WP*	0.683	0.4208
*Material × TFR*	0.132	0.8771
*Material × AMFR*	1.341	0.2895

**Table 7:** ANOVA of the influence of process parameters on Ra for each material and cutting region.

	PLA-CF		PLA	
*Effect*	*F*	*p*	*F*	*p*
*Zone*	42.18	**<0.001**	58.73	**<0.001**
*WP*	2.31	0.118	9.84	**0.008**
*TFR*	5.74	**0.021**	11.92	**0.004**
*AMFR*	1.12	0.342	1.43	0.266
*Zone × WP*	1.67	0.214	4.12	**0.038**
*Zone × TFR*	4.96	**0.029**	6.55	**0.012**
*Zone × AMFR*	0.88	0.462	1.77	0.196

**Table 8:** ANOVA of the influence of process parameters on Ra for each material and cutting region.

*Effect*	*F*	*p*
*Material*	24.67	**<0.001**
*Zone*	71.25	**<0.001**
*Material × Zone*	5.93	**0.006**

**Table 9:** ANOVA of the influence of process parameters on kerf taper for each material.

	PLA-CF		PLA	
*Effect*	*F*	*p*	*F*	*p*
*WP*	96.157	**0.000606**	28.815	**0.00582**
*TFR*	15.268	**0.0134**	0.302	0.7547
*AMFR*	16.231	**0.0120**	10.035	**0.0276**
*WP × TFR*	1.323	0.3623	4.543	0.0934
*WP × AMFR*	1.134	0.4073	2.812	0.1727
*TFR × AMFR*	1.211	0.4288	3.830	0.1108

**Table 10:** ANOVA of the influence of material on kerf taper.

*Effect*	*F*	*p*
*Material*	1.942	0.1825
*WP*	35.739	**0.000019**
*TFR*	2.426	0.1202
*AMFR*	8.547	**0.0030**
*Material × WP*	0.187	0.6714
*Material × TFR*	0.998	0.3906
*Material × AMFR*	0.277	0.7616
*WP × TFR*	0.668	0.5267
*WP × AMFR*	0.825	0.4559
*TFR × AMFR*	0.529	0.7161

## Data Availability

The original contributions presented in this study are included in the article. Further inquiries can be directed to the corresponding authors.
